# An Evolutionary Dynamics Model Adapted to Eusocial Insects

**DOI:** 10.1371/journal.pone.0055159

**Published:** 2013-03-01

**Authors:** Louise van Oudenhove, Xim Cerdá, Carlos Bernstein

**Affiliations:** 1 Université de Lyon, Lyon; Université Lyon 1; CNRS, UMR5558, Laboratoire de Biométrie et Biologie Evolutive, Villeurbanne, France; 2 Estación Biológica de Doñana, Consejo Superior de Investigaciones Científicas, Sevilla, Spain; University of Sussex, United Kingdom

## Abstract

This study aims to better understand the evolutionary processes allowing species coexistence in eusocial insect communities. We develop a mathematical model that applies adaptive dynamics theory to the evolutionary dynamics of eusocial insects, focusing on the colony as the unit of selection. The model links long-term evolutionary processes to ecological interactions among colonies and seasonal worker production within the colony. Colony population dynamics is defined by both worker production and colony reproduction. Random mutations occur in strategies, and mutant colonies enter the community. The interactions of colonies at the ecological timescale drive the evolution of strategies at the evolutionary timescale by natural selection. This model is used to study two specific traits in ants: worker body size and the degree of collective foraging. For both traits, trade-offs in competitive ability and other fitness components allows to determine conditions in which selection becomes disruptive. Our results illustrate that asymmetric competition underpins diversity in ant communities.

## Introduction

A fundamental challenge in community ecology is to understand the mechanisms allowing species coexistence [1]–[5]. In ant communities, competition for resources (e.g., nest sites and food) is an important force in structuring the community [6], [7], and interspecific differences at both morphological and behavioral levels represent important mechanisms of coexistence [8]. In many ecosystems, coexisting ant species differ in worker body size and colony foraging strategy [9]–[13]. In some Mediterranean ant communities, for instance [12], [13], mean worker body size ranges from 1.6 mm to 10.0 mm across species. Moreover, species exploit food resources differently. Some species forage individually: foragers that discover a food resource do not share information about resource location with nestmates. Other species forage collectively by recruiting nestmates to the food resource. The level of cooperation that characterizes collective foraging largely depends on the kind of signal involved in communication between nestmates (from antennal contact to long-lasting pheromone).

Trade-offs are frequently invoked to explain species coexistence [14]–[16]. At the evolutionary time scale, they drive niche differentiation processes, and contribute to the emergence of diversity [17], [18]. Several trade-offs have been identified in ant communities, such as the trade-off between competitive dominance and discovery abilities [10], [19], the trade-off between competitive dominance and thermal tolerance [12], [20], [21], and the trade-off between competitive ability and vulnerability to parasitoids [22], [23]. The so-called dominance-discovery trade-off refers to the negative correlation between the ability to defend food resources and the ability to find them and is thought to promote species coexistence [23], [24]. The general aim of this paper is to show how simple trade-offs, such as the dominance-discovery trade-off, can lead to the emergence and persistence of diversity in ant communities.

Adaptive dynamics theory [25]–[28] is a conceptual framework that can be used to model the long-term dynamics of evolutionary processes, specifically by analyzing the frequency-dependent evolution of quantitative traits (or strategies). This approach allows the modeling of phenomena such as evolutionary branching, during which a trait is driven to a value where selection becomes disruptive and thus the trait splits into two trait values that diverge gradually [27], [29]. Adaptive dynamics is consequently an efficient theoretical tool with which to test the emergence of diversity. Adaptive Dynamics is an approximation based on i/mutations are rare so that strategy dynamics at the evolutionary timescale is a reflection of population dynamics at the ecological timescale, and ii/reproduction is clonal: the only source of differentiation between generations is mutation. This second assumption is obviously not valid in ants, where most species reproduce sexually. Nevertheless, if mutations are assumed to be rare and to occur at a single locus, models using clonal reproduction versus random mating in monomorphic diploid populations yield similar conclusions regarding the occurrence of evolutionary branching [30], [31].

The challenge of modeling social insect colony fitness was first taken up by Macevicz and Oster [32]. They proposed a model in which a colony comprises two subunits: workers and sexuals (or reproductives). The colony life cycle is divided into an ergonomic phase (production of workers) and a reproductive phase (production of sexuals). During the ergonomic phase, the colony produces workers until they reach a threshold number. Then, during the reproductive phase, the energy that has been collected by workers is channeled into the production of sexuals. Colony fitness depends entirely on the production and success of reproductive adults. This model fits with most ant colony cycles, in that sexual production occurs annually and over a short time period that does not overlap with worker production [8], [33]. However, this modeling approach does not take into account potential reproductive conflicts between individuals within the colony, such as queen-worker or worker-worker conflicts over male parentage. In eusocial Hymenoptera, worker reproduction is prevented by behavioral mechanisms such as queen-policing, worker-policing, and self-restraint, making worker reproduction relatively rare in queenright colonies [34]–[37]. The resolution of conflicts among the lower-level units thus promotes the integrity of the higher-level unit: the colony. Ant colonies can be considered to be superorganisms [38]: individuals whose reproductive success determines the evolutionary outcome [7]. Furthermore, non-reproductive, worker-based behaviors, such as foraging, are expected to maximize overall energy intake for the colony [39]. In this context, representing the colony as a single unit and focusing on traits such as foraging strategy or worker size seems to be a reasonable approach. In this study, we employ this approach, ignoring any potential impacts of internal reproductive conflicts.

In ant communities, coexisting species display differences in morphology and foraging behavior. However, the factors underpinning the evolutionary processes that drive such trait differentiation are poorly understood. The goals of the current paper are to: i/propose a theoretical model that would enable the identification of the factors allowing the emergence and maintenance of diversity in ant communities, and ii/use this model to study the diversity of both foraging strategies and worker body sizes. The model links colony population dynamics to evolutionary processes by applying the adaptive dynamics framework to ants. The basic idea stems from the model of Macevicz and Oster [32]: the whole colony is divided into two subunits, reproductives and workers. Worker production is considered to be much faster than colony reproduction. This assumption of different timescales allows us to deterministically estimate the direction and speed of the evolution of the whole colony using worker population size. We applied this approach to study the evolutionary dynamics of two specific traits: worker body size and foraging strategy [9]. We examined the extent to which trade-offs might drive the evolution of these traits.

## General Model

We propose an adaptive dynamics model of the phenotypic evolution of eusocial insects. This model is limited to clonal reproduction. Phenotypic variation stems from mutations. Mutations are assumed to be rare so that population dynamics attains its population dynamical attractor between mutation events.

### Resident Community Model

We considered a community composed of a single monomorphic population characterized by a quantitative scalar trait *x*. Each colony of eusocial insects represents an individual. Colony abundance is denoted by *q* (*q* for queen). Colony’s size (i.e. the number of workers within colonies) is designated as *w* (*w* for worker). The demography of population *q* (i.e. the number of colonies) depends on the interactions of the colonies with their surrounding environment. As the interacting agents are the workers, the colony growth rate depends on the number of workers per colony. Let the function 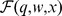 be the colony production rate. Colony population dynamics occurs at a longer timescale (*t*), whereas within colony dynamics, i.e. that of the worker population 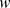, occurs at a shorter timescale (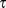). Let the function 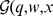) be worker production rate.

The dynamics of the community is described by the system (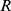):
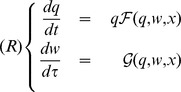



Colony dynamics occurs at a far longer timescale than those of worker production. This means that, during worker production, the number of colonies can be regarded as constant. We assume that worker population dynamics admits a stable and strictly positive equilibrium 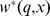. Therefore, workers are produced until a stable equilibrium 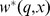 is reached.

If we focus on the system€s slower dynamics, the growth rate of the population of colonies is described by the function 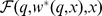. We assume that the population€s dynamics has a globally stable and strictly positive equilibrium 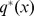. In a monomorphic community with trait 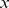, the population abundance is assumed to be constant at its equilibrium 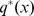 with number of workers 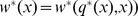.

### Resident-Mutant Community Model

We now consider a community composed of a resident monomorphic population, characterized by continuous trait 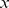, and a mutant population, with trait 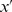. The abundance of residents and mutants is 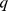 and 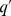, and a colony’s size (i.e. the number of workers in each kind of colony) is 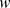 and 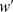, respectively. The community’s demography is described by the following system:
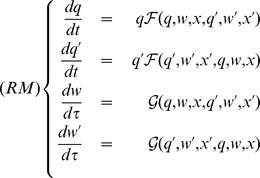



Colony population dynamics occurs at a far slower timescale. As in the monomorphic case (Resident model 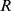), both 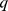 and 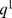 can be regarded as constant when considering faster system dynamics. We assume that the worker system has a stable and positive equilibrium 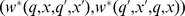. Before any change in the colony dynamics occurs, the worker system first reaches its stable equilibrium.

The slow-dynamics system (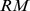) is thus given by:
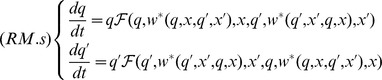



### Evolutionary Model

In the absence of mutants, the Resident-Mutant model degenerates into the Resident model: 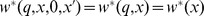.

We assume that, prior to the emergence of any mutant population, the resident population is constant at its equilibrium 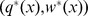. When a mutant with trait 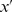 slightly different from the resident trait 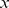 enters the community, it can either disappear or invade. The fate of mutants is determined by their invasion fitness 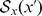, which represents the per capita growth rate of a very scarce mutant population in a community composed exclusively of residents:
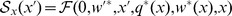
where 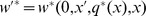 represents the stable equilibrium reached by the worker population in the scarce colonies of the mutant population.

Since mutations have small phenotypic effect, a mutant’s fitness may be linearized in the vicinity of the resident’s strategy. The evolutionary dynamics is then predicted by the local fitness gradient 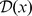 [29], defined as:
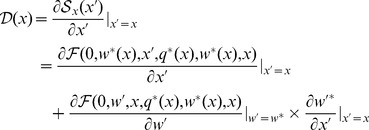
(1)


Strategies for which the local fitness gradient is zero are called singular strategies [27] (ss). The implicit function theorem allows us to express the partial derivative of worker equilibrium 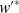 in terms of function 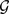 (File S1). Conditions under which a strategy is singular can thus be fully determined with 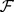 and 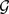. To simplify the equation, partial derivatives are denoted with superscripts (e.g. 
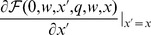
 is noted 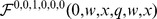, and 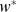 and 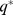 denote 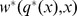 and 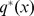.
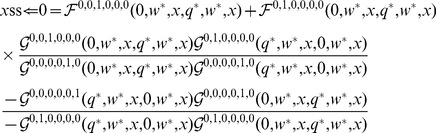
(2)


The evolution of a given trait in the monomorphic community depends on the convergence and stability of these singular strategies. On the one hand, a convergent stable strategy will be gradually reached via small evolutionary steps [40]. On the other hand, singular strategies that cannot be invaded by any proximate mutants are called evolutionarily stable strategies [41]. The property of evolutionarily stability depends only on the second derivative of the invasion fitness function with respect to the mutant strategy while the property of convergence stability depends also on the second derivative of the invasion fitness with respect to both the mutant strategy and the resident strategy [27], [29]. In this model, they can be determined according to 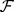 and 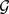. However, since the explicit conditions consist of extremely long expressions, we do not detail the general case here.

Depending on the convergence stability and the evolutionary stability of a singular point, different evolutionary scenarios unfold [28]. For our purpose, two scenarios are of particular interest: i/singular strategies that are both convergence stable and evolutionary stable are evolutionary end points (sometimes called continuously stable strategy in the literature); ii/singular strategies that are convergence stable but that can be invaded by nearby mutants are known as evolutionary branching points [27], [29]. In this case, when the monomorphic population reaches this strategy, it experiences disruptive selection and splits into two diverging subpopulations.

The evolutionary scenario of this dimorphic community can be deduced from the analysis of evolutionary isoclines [42]. These lines divide the set of possible two-strategy coexistence states into a number of regions with different coevolutionary directions. They consist of pairs of traits at which the invasion gradient of scarce mutants in a community with two resident traits equal to zero. To establish the evolutionary isoclines, the community with two traits 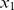 and 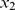 is considered to be at equilibrium. Let 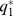 and 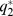 be the equilibrium number of colonies associated with each strategy, and 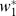 and 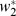 their respective colony’s size. As in the monomorphic case, the fate of scarce mutants is determined by their per capita growth rate in the resident community:
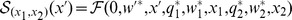
where 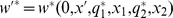 represents the stable equilibrium reached by the worker population in the mutant colonies.

## Illustrating the Model

To illustrate this model, we present two specific examples of the evolutionary dynamics of ant traits. The first example deals with worker body size, a morphological trait. The second example deals with the degree of cooperative foraging, a behavioral trait.

For both examples, colony and worker production rates were specified with explicit functions. First, singular strategies and their properties were identified analytically both in the general case and by assigning a particular shape to their functions (Table 1). Then, numerical calculations were used to predict the evolutionary scenario that emerged in the monomorphic population as a result of parameter values. Finally, in the case of a branching event, evolutionary isoclines were drawn to determine the dynamics of a community with two evolving strategies.

**Table 1:** Mathematical conditions identifying the singular strategies and their properties of evolutionary stability and convergence.

Example 1: evolution of worker body size			
General case			
a/	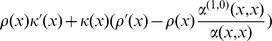	=	0
b/	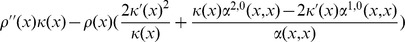	<	0
c/	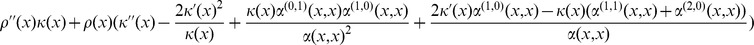	<	0
Specific functions			
a/	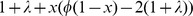	=	0
b/	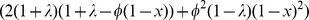	>	0
c/	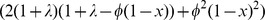	>	0
**Example 2: evolution of foraging strategy**			
General case			
a/	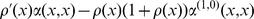	=	0
b/	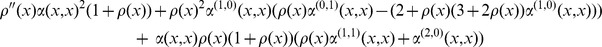	<	0
c/	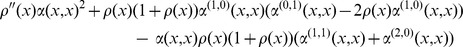	<	0
Specific functions			
a/	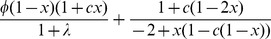	=	0
b/	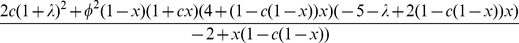	<	0
c/	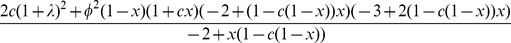	<	0

Strategy properties of the different models as a function of the convexity of the per worker intrinsic growth rate 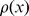, the competitive kernel 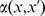, and the colony’s size at carrying capacity 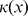. In both examples, mathematical conditions have been simplified assuming 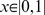 and depend on competition intensity 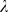, competition asymmetry 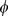, and parameter 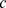. a/conditions for x to be a singular strategy; b/condition for x to be an evolutionary stable strategy; c/conditions for x to be a convergent stable strategy.

To test the robustness of the predictions based on the adaptive dynamics approximation and delineate the conditions under which a high degree of polymorphism can evolve, stochastic simulations were performed. The simulations started with a monomorphic community. At each evolutionary time step, a new mutant appeared. Mutations were drawn from a truncated normal distribution with a fixed variance and a mean given by the trait value of the parent. Mutants were introduced at a low initial frequency ( = 1%). The resident-mutant community varied at the ecological timescale according to the population dynamics specified by system (RM). The within-colony dynamics was set to be 100 times faster than the colony dynamics. Strategies whose abundance dropped below a threshold (frequency 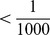) were eliminated. The remaining strategies defined the new resident community in the next evolutionary step.

Analyses were performed with Wolfram *Mathematica* 8.0 software.

### Evolutionary Dynamics of Worker Body Size

Let a community be composed of 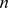 strategies. Let strategy 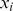 be the mean worker body size. Let 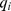 and 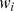 be the number of colonies and the colony’s size associated with the strategy 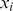, respectively. The colony production rate 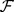 is represented by a Lotka-Volterra competition model. It incorporates the per worker colony intrinsic growth rate 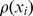 weighted by the number of workers 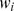, the effect of intraspecific competition 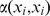 weighted by the number of conspecific workers 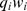, and the effect of interspecific competition 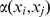 weighted by the number of workers of other species 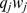 (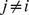). Overall, the effects of intra- and interspecific competitive interactions on 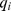 growth are 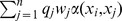. The worker production rate 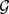 is a logistic growth equation. Since both worker and colony production depends on the energy entering the nest, 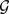 is also proportional to the intrinsic colony growth rate 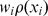. In this example, we assume that the production of workers is independent of the interaction with neighboring colonies. However, we also assume that total worker biomass is limited in the nest, leading to a negative correlation between the maximum number of workers and their size. Function 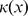 (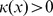) represents colony’s size at carrying capacity. The trade-off between the number of workers and worker body size is translated mathematically by the assumption 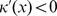.
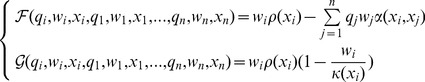
(3)


The general framework presented above enables us to identify the singular strategies and their evolutionary properties according to the shape of both 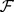 and 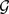. The establishment of such properties with the functions specified in system (3) allows us to then classify these singular strategies according to the shape of functions 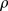, 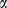, and 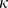 (Table 1).

Let 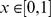 be the mean worker body size. The boundary is fixed arbitrarily, and low values of 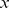 imply that workers are small while high values signify that workers are large. Assuming that worker loading capacity increases with body size [43], we suggest that the per worker intrinsic growth rate is an increasing function of 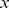. Here, we choose the linear function
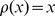



**Figure 1:**
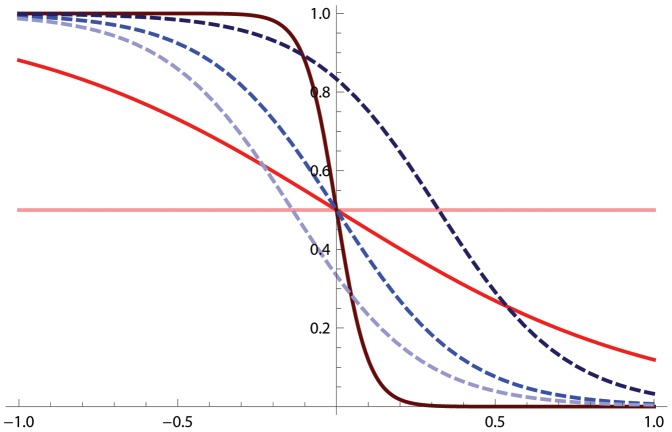
Function 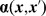 as a function of 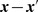. Red full lines show how increasing competition asymmetry affects 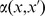: 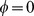, light red; 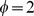, red, 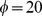, dark red (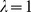 for all red lines). Blue dashed lines show the effect of competition intensity: 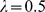, light blue; 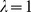, blue; 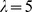, dark blue (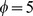 for all blue lines).

Likewise, we assume that larger body size results in a competitive advantage. A sigmoidal function was used for 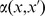 [30], [44] (Fig. 1).
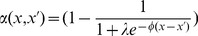
(4)


This function models the effect of an 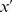-colony on an 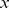-colony. Parameters 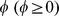 and 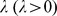 shape the strength of competition. Competition asymmetry 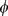 only operates on interspecific competition and emphasizes the difference between strategies: the higher the value, the more important a small difference in strategies will be. On the other hand, 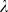 applies to both intra- and interspecific competition and will be referred to as competition intensity.

**Figure 2:**
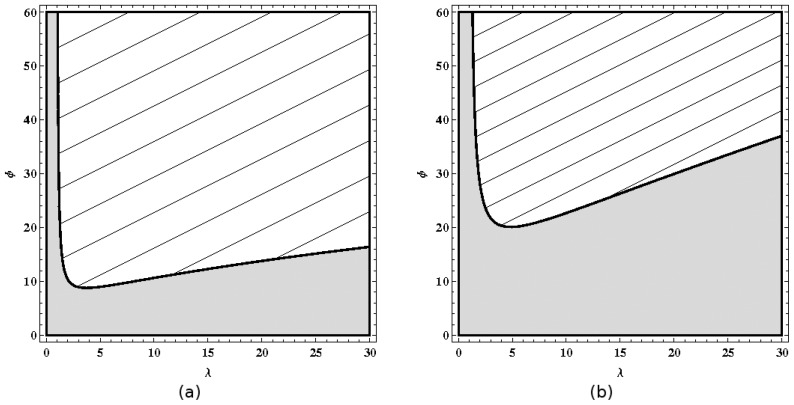
Evolutionary outcomes of life history traits (a: worker body size, b: degree of cooperative foraging) as a function of the interplay between competition intensity 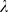 and asymmetry 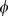. Singular strategies are color-labeled according to their evolutionary properties. Parameter pairs leading to an evolutionary ending point are in gray (both Ess and Css) and the dashed surface corresponds to evolutionary branching points (Css, but not Ess). Note that competition intensity cannot be null (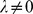): without competition, the resident population would not be limited (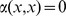) and would tend to infinity, thus the assumption of a stable resident population would be transgressed.

We assume a negative correlation between colony’s size carrying capacity and worker body size. We used the linear function:
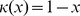



The evolutionary scenario can be predicted from the stability and convergence of the singular strategies (Table 1). Long-term evolution depends on the interplay between both competition intensity 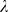 and competition asymmetry 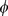 (Fig. 2.a). Two scenarios occur. First, if either competition intensity or competition asymmetry is weak (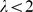 or 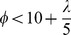 in Fig. 2.a), long-term evolution results in a single monomorphic strategy. This strategy is called the evolutionary end point [45]. Second, if both competition intensity and competition asymmetry are strong (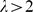 and 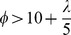), the system evolves toward an evolutionary branching point: directional evolution turns into disruptive selection, and the resident community becomes dimorphic.

**Figure 3:**
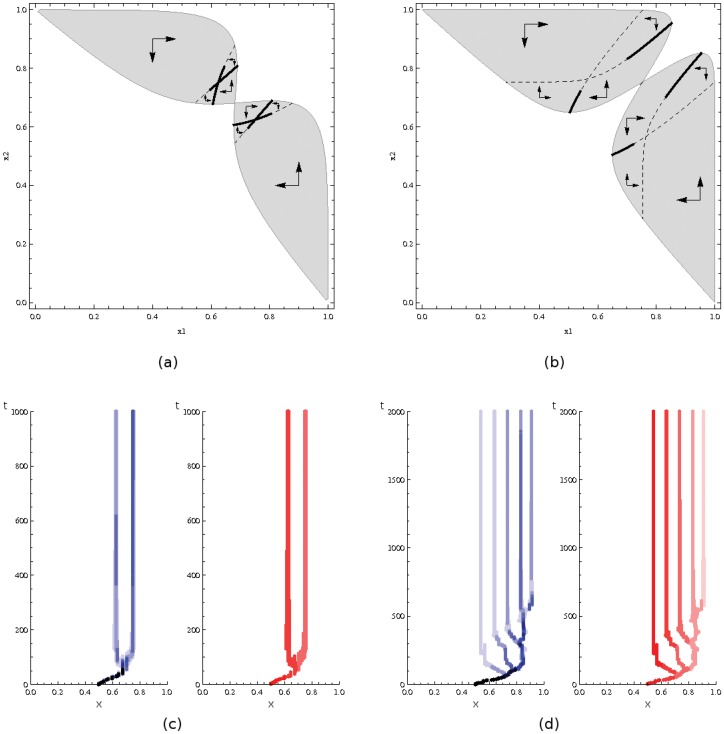
Evolution of worker body size predicted by (a-b) evolutionary isoclines and illustrated by (c–d) simulated evolutionary trees: a,c: stable dimorphism (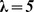, 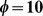), b,d: increasing levels of polymorphism as a result of repeated branching events (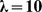, 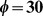). (a–b) Shaded areas are regions of possible coexistence between strategies 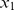 and 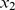. Arrows indicate the direction of evolution. Thick (resp. dashed) isoclines represent fitness maxima (resp. minima). (c–d) Blue trees represent the relative numbers of colonies using a given strategy (the darker the blue, the more common the strategy). Red trees represent the associated colony’s size (the darker the red, the more workers present in the colony). Simulations start with a monomorphic community of medium-sized workers (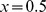). Genetic variance is estimated to be 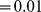. The ecological timescale is assumed to be 1000 times faster than the evolutionary timescale.

The fate of the dimorphic community emerging from the branching event depends on the convergence and stability of the evolutionary isoclines (Fig. 3. (a,b)). After a branching event, there are two possible outcomes. First, if competition is intermediate (intermediate values of 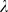 and 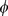), the dimorphic community reaches a stable coalition, and the two equilibrium strategies coexist. Second, if competition is strong (high values of 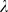 and 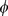), the dimorphic community evolves toward a convergent singular coalition of strategies that is evolutionarily unstable, predicting the occurrence of more branching events. The resident community will reach higher levels of polymorphism.

These predictions were confirmed by our simulations (Fig. 3. (c,d)). In the case of intermediate competition (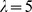, 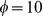), the two strategies stably coexist in the community. Colonies with big workers are more abundant but contain fewer workers than colonies with small workers. In the case of strong competition (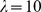, 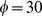), the community undergoes four branching events, reaching a stable equilibrium of five different body sizes. As in the former case, bigger workers mean more abundant but smaller colonies. The number of branching events depends on the intensity and asymmetry of competition: higher values of 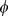 and larger differences between 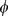 and 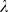 tend to increase the number of coexisting separate branches in the simulated evolutionary tree. During the simulations, the difference between slow and fast timescales can be relaxed by letting worker production dynamics to be as fast as colony production dynamics. The simulation results are qualitatively similar: the evolutionary scenarios are identical but occur much more slowly.

### Evolutionary Dynamics of Foraging Strategies

In this example, the worker production rate is no longer restricted by the strategy-dependent colony’s size at carrying capacity. We assume that worker production suffers as a result of competition with neighboring colonies in accordance with a Lotka-Volterra competition model. The equation for worker production 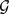 thus includes the intrinsic colony growth rate 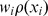, and a carrying capacity scaled to 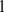 implying that worker production is restrained both by itself and by the competitive weight of the neighboring colonies. The equation for colony production rate 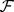 remains the same as previously.
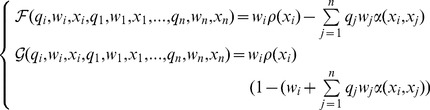
(5)


Let 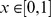 be the degree of cooperation between workers during foraging. Low values of 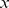 denote weak cooperation. For instance, 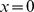 would represent strict individual foraging. Conversely, high values stand for highly collective strategies such as mass recruitment. We consider that 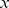 underpins the dominance-discovery trade-off [24]: at high values of 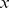 (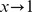), the colony is a good competitor but a poor discoverer. Conversely, at low values of 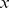 (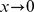), the colony is a weak competitor but good at resource discovery. The per worker intrinsic growth rate 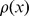 takes into account both food discovery and food exploitation. The ability to discover food rapidly is negatively correlated with the degree of collective foraging. However, the ability to exploit a food resource might increase with collective foraging, depending on food type. To model the per worker intrinsic growth rate, we use the following polynomial:
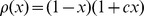
where 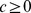 represents the advantage of cooperative foraging in food exploitation. We can imagine, for instance, that if resources are of small size, recruiting nestmates to them does not present any advantage, and 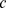 would thus tend to 0. On the other hand, if food resources are large, recruitment might accelerate the exploitation process, and the value of 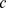 would be high. Note that, for 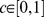, 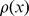 is maximized by 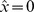 (individual foraging); and for 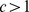, the per worker intrinsic growth rate is maximized by an intermediate degree of cooperation 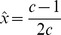. Function 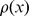 is thus an unimodal function that represents how the degree of cooperation increases the food exploitation process but decreases the food resource discovery. 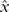 would be the optimal strategy in a community without competition (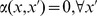).

When colonies interact with each other, the advantage conferred by cooperation depends on the strategies of the two competitors: the larger the difference between 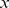 and 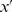, the lower the impact of 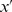-colonies on 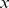-colonies and the higher the impact of 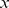-colonies on 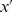-colonies. The same function as was used previously was employed to model the effect of 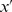-colonies on 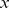-colonies (Eq. 1).

The evolutionary scenario can be predicted from the conditions for convergence and stability at a singular point (Table 1). Two different patterns are possible and depend on the competition parameters (Fig. 2.b). If competition intensity or competition asymmetry is weak (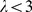 or 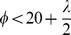 in Fig. 2.b), the unique equilibrium of the evolutionary dynamics is an end point: the monomorphic community evolves until reaching a strategy that cannot be invaded by any nearby mutants. If both competition intensity and competition asymmetry are strong (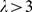 and 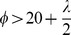), the singular strategy loses its evolutionary stability. Evolution leads the monomorphic community to this value, and selection then becomes disruptive. After this branching event, two strategies appear and diverge from each other.

**Figure 4:**
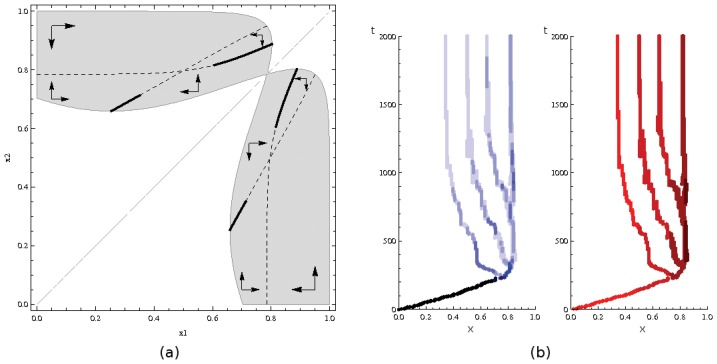
Evolution of intensity of cooperative foraging predicted by (a) evolutionary isoclines and illustrated by (b) simulated evolutionary trees: the initially monomorphic community undergoes several branching events and becomes highly polymorphic. (a) Shaded areas represent regions of possible coexistence between strategies 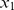 and 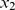. The direction of evolution is indicated by arrows. Stable (resp. unstable) isoclines are represented by thick (resp. dashed) lines. (b) The blue tree (left) represents the relative number of colonies using the corresponding strategy (the darker the blue, the more common the strategy), and the red tree (right) represents the associated colony’s size (the darker the red, the more workers present in the colony). Simulations start using individual foraging as the ancestral strategy (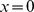). Parameter values: 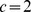, 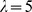, 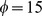. (See Fig. 3 for simulation details).

The structure of the evolutionary isoclines allows us to determine the dynamics of the two strategies after the branching event. According to the arrows indicating the direction of evolution (Fig. 4.a), one of the strategies increases while the other decreases. The evolutionary isocline towards which the two strategies evolve loses its evolutionary stability (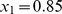, 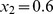 in Fig. 4.a), thereby causing a second branching event. The community then reaches a higher degree of polymorphism.

Stochastic simulations confirm this pattern (Fig. 4.b). As predicted, the monomorphic community first evolves to a branching point and becomes dimorphic. It then undergoes a second branching event followed by a third. Four different strategies coexist in the community. Colonies using the most collective strategy (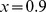) are slightly more abundant than others. Simulations allow us to determine the number of workers associated with each strategy. Colony’s size appears to be positively correlated with the degree of cooperation in foraging. Parameter 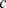 affects the value of coexisting strategies. If the advantage of collective foraging is low with respect to the per worker intrinsic growth rate 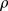 (e.g., for 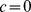), coexisting strategies are distributed between 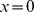 and 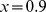. Higher 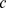 values increase the value of the lesser-valued strategies. For instance, for 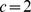, strategies are distributed between 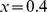 and 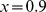. The number of coexisting strategies depends on the intensity and asymmetry of competition: Increasing 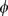 or 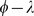 augments the number of branching events. Simulations in which the difference between timescales was relaxed show that the results are qualitatively equivalent, but that more evolutionary time is needed to produce the patterns.

## Discussion

This work presents a model that can be used to study the evolutionary dynamics of life history traits or specific behaviors in eusocial insects. Two examples that consider the evolutionary dynamics of worker body size and foraging strategies in ants are developed. They show how simple trade-offs can explain the emergence and maintenance of different strategies in a community depending on the interplay between intra- and interspecific competition.

This work shows how adaptive dynamics theory can be applied to eusocial insect societies. The classic model of Macevicz and Oster [32] considers a colony as being divided into workers and sexuals and defines fitness as the rate of sexual production. Our model is inspired by this work but instead considers insect societies in a long-term evolutionary perspective. In the majority of ant societies, workers do not reproduce [34]–[37], and thus the ant colony can be regarded as an individual, whose reproductive success determines the evolutionary outcome for the species [7]. Therefore, we assume that selection exclusively acts at the level of the whole colony, with colony fitness depending on its size, i.e. its number of workers. The simplifying assumptions are that colony development (i.e. worker production) is a faster process than colony reproduction (i.e. production of new colonies). Colony development and reproduction are defined by a slow-fast system of differential equations that represent colony population dynamics. Interactions between different colony populations define the community’s dynamics. This dynamics, which operates at the ecological timescale, drives the evolutionary process at the evolutionary timescale. The model relies on a strong assumption: clonal reproduction. In ants, this is the exception rather than the rule (but see [46]–[50]), and it raises some questions about our results. In particular, to what extent can conclusions from clonal models of adaptive dynamics be applied to populations of sexual organisms? The conditions for evolutionary branching derived from clonal populations also apply to sexual population (under some conditions: for a discussion of this topic see [51, Section 2.6]), meaning that scenarios leading to the selection of a single strategy before the branching event occurs, are preserved with sexual reproduction. However, once evolutionary branching occurs, random mating and recombination might recreate intermediate phenotypes and thereby prevent the emergence of discrete cluster. Thus, different mechanisms creating phenotypic variation due to evolutionary branching exist [52]. In one of the scenarios, disruptive selection could favor assortative mating and therefore improve the degree of reproductive isolation [31]. In this case, results from the clonal model are restored, and trait diversification from branching events might even lead to sympatric speciation.

The first version of our model incorporates a trade-off between worker size and number, by considering that they were negatively correlated. At the colony level, this becomes a trade-off between colony size and competitive ability. The intrinsic growth rate (i.e. the growth rate of colonies assuming neither intra- or inter-specific competition) is maximized by medium sized worker (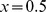). However, these colonies are outcompeted by colonies with larger worker (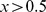). This model predicts different evolutionary outcomes. When competition asymmetry is strong relative to competition intensity, the model predicts the emergence of species with different mean worker body sizes in the community. This prediction fits with observations of natural communities, in which differences in worker body size are associated with resource subdivision [9]. For instance, in harvester ants, worker body size is positively correlated with the size of the seeds the species preferentially collects [9], [53]–[55]. For simplicity’s sake, we only considered the mean worker body size in the colony. However, in social insects, castes are a fundamental part of colonial organization [39]: polymorphism promotes the division of labor. With regards to body size, colony polymorphism enhances diet breadth [54] and is negatively correlated to species diversity in the community [53]. To gain a better understanding of the evolution of worker body size, it might thus be important to include a high degree of colony polymorphism by differentiating workers by caste or size class. Functional specialization might be favored by natural selection, depending on the traits involved in different tasks, and their respective performance efficiency [56]. Size polymorphism within a colony could thus evolve if the gain in performance associated with the different sizes is high enough.

The second trait considered in our model is the foraging system. Since collectively foraging species are superior at interference competition, we could have expected that strong competitive pressure would favor the evolutionary stability of collective foraging strategies. In contrast, collective foraging also seems to be favored when competition is weak. On the other hand, when the overall level of competition intensity (both intra- and inter-specific) is strong relative to inter-specific competition, the initially monomorphic community becomes polymorphic, with different degrees of collective foraging coexisting. Regardless of the ancestral strategy, foraging evolves to become collective. However, when all colonies adopt this strategy, selection becomes disruptive, and negative frequency-dependent selection favors the appearance of divergent strategies. The stable community composition at the end of the evolutionary process depends on the strength of competition. Generally, the stronger the effect of competition, the higher the number of coexisting strategies. Furthermore, in environments in which collective foraging does not afford a distinct advantage in exploiting food resources, coexisting strategies might vary from completely individual to highly collective, as occurs in some Mediterranean ant communities that include foraging strategies such as individual foraging, tandem running, group recruitment, and mass recruitment [12]. Indeed, in those communities, the performance of collectively foraging species might be reduced because of high temperatures [57], which would allow the whole range of foraging strategies to co-evolve and coexist. In summary, even a simple model might explain the appearance and coexistence of foraging strategies involving different degrees of collective behavior.

Many studies on the optimality of collective foraging behavior agree on the importance of the number of individuals and conclude that collective foraging is optimal for large colonies [58]–[60]. Since a positive correlation between colony size and the degree of communication in ant foraging behavior has been established [61], the trend seems to be that each colony’s size corresponds to a given optimal foraging strategy. A model incorporating an explicit function for worker production relaxes the need to make preliminary assumptions about colony’s size. Furthermore, colony’s size can instead be predicted as an evolutionary outcome. When worker production depends on competitive interactions with neighboring colonies, the predicted colony’s sizes are positively correlated with the degree of cooperation characterizing the foraging strategies. Indeed, foraging with more collective strategies increases competitiveness, which in turn increases colony growth. This model can thus serve as a heuristic basis to illustrate the co-evolution between foraging strategy and colony’s size.

Both examples presented emphasize the importance of competitive trade-offs for the emergence and coexistence of different strategies within the same community. Trade-offs between competitive abilities and other fitness components such as mortality [62], colonization capacity [14], [63], or resource exploitation [15], [24] are often suggested as an explanation for species coexistence. Furthermore, the emergence of different species (or sets of individuals using a given strategy) can be made possible by the degree of competition asymmetry present in such trade-offs [30], [44]. In both models presented in this work, more intense competition enhanced community diversity. In fact, highly competitive interactions made selection disruptive, and thereby provoked strategy divergence. In these examples, competition asymmetry is a requisite for strategy divergence. If competition were symmetric, there would not have any branching event. In these models, highly asymmetric competition means that two populations with slightly different strategies have very different effect on the growth rate of the other population. Conditions for strategy divergence appear when the gain in competitive ability is balanced by a loss in intrinsic growth rate. This strategy divergence resulted in specialization in either a morphological (i.e. worker body size) or a behavioral (i.e. foraging strategy) trait. Competition drives niche shift and character displacement until the community stabilizes. In the example on worker body size, the different niches at equilibrium might be reduced to either i/few colonies of many medium sized workers that maximize their intrinsic growth rate, but present low competitive ability; or ii/many colonies of few large workers with a lower intrinsic growth rate, and high competitive ability. Similarly, in the example on foraging strategies, colonies invest either in i/high intrinsic growth rate per worker with a low number of workers per colony (implying low intra-specific competition) and a low competitive ability for inter-specific interactions; or in ii/lower intrinsic growth rate per worker with many workers in the colony (meaning high intra-specific competition) and a high competitive ability. The model thus allows us to assess the ghost of competition past [64], which may be responsible for community structure in resource-limited ant communities [9].

This study focuses on social insects and considers the colony to be the unit of selection. Natural selection is modeled by assuming that the fitness of colonies depends explicitly on the energy accumulated by workers. This approach could be extended to the study of evolutionary processes in other social systems that comply with the following assumptions: i/reproduction can be represented as being carried out by a collective entity; ii/the reproduction of this collective entity occurs at a much slower rate than the production of its interacting agents.

## Supporting Information

File S1 **The implicit function theorem applied to workers production allows to explicit analytical expressions of the partial derivatives of worker equilibrium.**
(PDF)
