# Supplementary material for: An Evolutionary Dynamics Model Adapted to Eusocial Insects
Source: PLoS One. 2013 Mar 1;8(3):e55159. doi: 10.1371/journal.pone.0055159 (PMC3585944; doi:10.1371/journal.pone.0055159)
Supplement: File S1 — The implicit function theorem applied to workers production allows to explicit analytical expressions of the partial derivatives of worker equilibrium. (PDF) [file pone.0055159.s001.pdf]

Supplementary Information:

An evolutionary dynamics model adapted to eusocial insects

Louise van Oudenhove, Xim Cerdá, Carlos Bernstein

1 The Implicit Function Theorem applied to workers production

Let g_i be real valued functions defined and continuously differentiable on an open set $\mathcal{D} \subset \mathbb{R}^6$. Let $(w_0, w'_0, q_0, q'_0, x_0, x'_0) \in \mathcal{D}$, such as

$$\begin{aligned} g_1(w_0, w'_0, q_0, q'_0, x_0, x'_0) &= 0 \\ g_2(w_0, w'_0, q_0, q'_0, x_0, x'_0) &= 0 \end{aligned}$$

Let Q be the Jacobian matrix

$$Q = \begin{pmatrix} \frac{\partial g_1(w,w',q,q',x,x')}{\partial w} & \frac{\partial g_1(w,w',q,q',x,x')}{\partial w'} \\ \frac{\partial g_2(w,w',q,q',x,x')}{\partial w} & \frac{\partial g_2(w,w',q,q',x,x')}{\partial w'} \end{pmatrix}$$

Further assume that, for $(w, w', q, q', x, x') = (w_0, w'_0, q_0, q'_0, x_0, x'_0)$, Q has rank 2 (i.e. $det(Q) \neq 0$).

Then there exists a neighborhood $N_\delta(w_0, w'_0, q_0, q'_0, x_0, x'_0) \subset \mathcal{D}$, an open set $\mathcal{W} \subset \mathbb{R}^4$ containing (q_0, q'_0, x_0, x'_0), and real valued functions W_1 and W_2 continuously differentiable on $\mathcal{W}$, such that

$$\begin{aligned} w_0 &= W_1(q_0, q'_0, x_0, x'_0) \\ w'_0 &= W_2(q_0, q'_0, x_0, x'_0) \end{aligned}$$

For every $z = (q, q', x, x') \in \mathcal{W}$ we have

$$\begin{aligned} g_1(W_1(z), W_2(z), z) &= 0 \\ g_2(W_1(z), W_2(z), z) &= 0 \end{aligned}$$

Furthermore for $z \in \mathcal{W}$, the partial derivatives of $W_i(z), i = 1, 2$ are the solutions of the set of linear equations given by

$$J = -Q^{-1}P$$

where P and J are the Jacobian matrix

$$P = \begin{pmatrix} \frac{\partial g_1(w,w',q,q',x,x')}{\partial q} & \frac{\partial g_1(w,w',q,q',x,x')}{\partial q'} & \frac{\partial g_1(w,w',q,q',x,x')}{\partial x} & \frac{\partial g_1(w,w',q,q',x,x')}{\partial x'} \\ \frac{\partial g_2(w,w',q,q',x,x')}{\partial q} & \frac{\partial g_2(w,w',q,q',x,x')}{\partial q'} & \frac{\partial g_2(w,w',q,q',x,x')}{\partial x} & \frac{\partial g_2(w,w',q,q',x,x')}{\partial x'} \end{pmatrix}$$

$$J = \begin{pmatrix} \frac{\partial W_1(q,q',x,x')}{\partial q} & \frac{\partial W_1(q,q',x,x')}{\partial q'} & \frac{\partial W_1(q,q',x,x')}{\partial x} & \frac{\partial W_1(q,q',x,x')}{\partial x'} \\ \frac{\partial W_2(q,q',x,x')}{\partial q} & \frac{\partial W_2(q,q',x,x')}{\partial q'} & \frac{\partial W_2(q,q',x,x')}{\partial x} & \frac{\partial W_2(q,q',x,x')}{\partial x'} \end{pmatrix}$$

To apply this theorem to our model, functions $g_i, i = 1, 2$ must be identified as follows

$$\begin{aligned} g_1(w, w', q, q', x, x') &= \mathcal{G}(q, w, x, q', w', x') \\ g_2(w, w', q, q', x, x') &= \mathcal{G}(q', w', x', q, w, x) \end{aligned}$$

The theorem thus allows to identify partial derivatives. For instance,

$$\begin{aligned} \frac{\partial w'^*}{\partial x'} &= \frac{\partial W_2(q^*,0,x,x')}{\partial x'} \\ &= \frac{\frac{\partial \mathcal{G}(0,w',x',q,w,x)}{\partial x'} \frac{\partial \mathcal{G}(q^*,w,x,0,w',x')}{\partial w} - \frac{\partial \mathcal{G}(q,w,x,0,w',x')}{\partial x'} \frac{\partial \mathcal{G}(0,w',x',q^*,w,x)}{\partial w}}{\frac{\partial \mathcal{G}(q,w,x,0,w',x')}{\partial w'} \frac{\partial \mathcal{G}(0,w',x',q^*,w,x)}{\partial w} - \frac{\partial \mathcal{G}(0,w',x',q,w,x)}{\partial w'} \frac{\partial \mathcal{G}(q^*,w,x,0,w',x')}{\partial w}} \end{aligned}$$
